# Silica-Decorated NiAl-Layered Double Oxide for Enhanced CO/CO_2_ Methanation Performance

**DOI:** 10.3390/nano12173041

**Published:** 2022-09-01

**Authors:** Wenxia Yan, Yangyang Li, Junming Zeng, Wentao Bao, Huanhuan Zhao, Jiangbing Li, Poernomo Gunawan, Feng Yu

**Affiliations:** 1 Key Laboratory for Green Processing of Chemical Engineering of Xinjiang Bingtuan, School of Chemistry and Chemical Engineering, Shihezi University, Shihezi 832003, China; 2 School of Chemical and Biomedical Engineering, Nanyang Technological University, Singapore 637459, Singapore; 3 Carbon Neutralization and Environmental Catalytic Technology Laboratory, Bingtuan Industrial Technology Research Institute, Shihezi University, Shihezi 832003, China

**Keywords:** CO/CO_2_ methanation, carbon neutrality, synthetic natural gas, layered double oxide, two-dimensional material

## Abstract

CO/CO_2_ hydrogenation has attracted much attention as a pathway to achieve carbon neutrality and production of synthetic natural gas (SNG). In this work, two-dimensional NiAl layered double oxide (2D NiAl-LDO) has been successfully decorated by SiO_2_ nanoparticles derived from SiCl_4_ and used as CO/CO_2_ methanation catalysts. The as-obtained H-SiO_2_-NiAl-LDO exhibited a large specific surface area of 201 m^2^/g as well as high ratio of metallic Ni^0^ species and surface adsorption oxygen that were beneficial for low-temperature methanation of CO/CO_2_. The conversion of CO methanation was 99% at 400 °C, and that of CO_2_ was 90% at 350 °C. At 250 °C, the CO methanation reached 85% whereas that of CO_2_ reached 23% at 200 °C. We believe that this provides a simple method to improve the methanation performance of CO and CO_2_ and a strategy for the modification of other similar catalysts.

## 1. Introduction

The CO/CO_2_ methanation reaction is the study of the synthesis of CH_4_ from the hydrogenation of CO/CO_2_ with H_2_ [1,2]. The methanation reaction can capture a large amount of CO_2_ and CO emitted from fossil fuel combustion, such as coke-oven gas, which can promote efficient conversion and utilization of CO_2_. In addition, it also facilitates carbon neutrality, and when combined with renewable H_2_, it can produce value-added chemicals and energy substitutes [3]. In particular, methanation reactions have widely received a lot of attention [4,5] as the generated synthetic natural gas can effectively relieve pressure on natural gas supplies [6,7]. Methanation catalysts are often based on Group VIII metals (e.g., Ru [8,9,10], Rh [11], Co [12,13], Fe [14,15], Ni [16,17]) that are supported on various oxide supports. Among the active metals used in methanation reactions, precious metals such as Ru and Rh are the most reactive and selective, but their relatively high cost makes them at the disadvantage economically. The use of Co-based catalysts for methanation reactions is also commonly studied, but their conversion and selectivity are slightly inferior to those of Ni-based catalysts. However, despite having better conversion rates than Ni, Fe has been little studied as an active center, probably due to its poor selectivity. As a consequence, nickel-based catalysts have become among the most widely studied materials, as their low cost, high activity and natural abundance make them more attractive for industrial-scale applications. [18]. Ni reacts with CO at a low temperature to form nickel carbonyl, and the high exothermic property of methanation renders Ni prone to carbon deposition and sintering at high temperatures. Therefore, the main problem to be solved by the methanation reaction is the poor low-temperature performance and particles sintering at high temperatures [19,20].

Hydrotalcite is a layered double hydroxide (LDH) material, where its layered structure can offer better exposure of catalytic active sites and may accommodate various anions that are homogeneously distributed in the interlayer gallery. It also exhibits highly tunable basic sites and has a good catalytic effect on CO/CO_2_ methanation reactions [21,22,23]. There are many methods to prepare hydrotalcite, such as coprecipitation [24,25], hydrothermal method [26], etc. Different preparation methods have their own advantages, which can affect the catalytic performance of the catalyst. For example, Zhang et al. [27] prepared highly stable Ni-based hydrotalcite using a self-sacrificial template method for CO_2_ methanation reaction. The catalyst formed a structure where Ni particles were embedded into the AlO_x_ matrix, which could minimize the agglomeration and sintering of Ni particles and improved the dispersion and stability of the catalyst. Ren et al. [20] exfoliated NiAl-LDH with an ethanol aqueous solution by ultrasonic treatment and embedded well-dispersed Ru nanoparticles onto the delaminated LDH. It was observed that the targeted activation of CO_2_ by the exfoliated LDH fraction was important in promoting the CO_2_ methanation reaction. Yao et al. [28] synthesized highly dispersed oxygen-enriched defective Ni catalysts with different NiAl molar ratios by flash nanoprecipitation (FNP) followed with delamination process in acetone. The low-temperature catalytic performance of the catalyst was found to be significantly improved, which may have been due to the large specific surface area and the defective surface of the catalyst obtained by stripping and FNP technology, respectively. The preparation method of the catalyst has an extremely important influence on dispersion and oxygen vacancy and ultimately affects the performance of the catalyst [29].

Herewith, we employed SiO_2_ nanoparticles derived from SiCl_4_ to decorate two-dimensional NiAl layered double oxide (2D NiAl-LDO) and improve its low-temperature CO methanation performance. First, the addition of SiO_2_ to the methanation catalyst improved and increased the specific surface area and dispersion of the catalyst [30,31]. Moreover, SiCl_4_ which is a by-product of the polysilicon industry is known to cause serious environmental pollution, and its treatment may pose a major ecological problem. Therefore, the re-use of silicon tetrachloride may help solve a major industrial problem [32]. In addition, we believe that it provides an additional strategy to easily improve low-temperature CO/CO_2_ methanation and shows potential for the application of similar catalysts.

## 2. Materials and Methods

### 2.1. Catalysts Preparation

Nickel acetate tetrahydrate (Ni(CH_3_COO)_2_·4H_2_O), aluminum nitrate nonahydrate (Al(NO_3_)_3_·9H_2_O), Na_2_CO_3_, NaOH and silicon tetrachloride (SiCl_4_) were purchased from McLean; all reagents are analytical grade.

For the methanation reaction, three samples were prepared, namely NiAl-LDH, SiO_2_-NiAl-LDH and SiCl_4_-NiAl-LDH, which indicate pristine NiAl-LDH, SiO_2_ and SiCl_4_ supported NiAl-LDH, respectively. In brief, 9.525 g Ni(CH_3_COO)_2_·4H_2_O and 4.785 g Al(NO_3_)_3_·9H_2_O were dissolved in 100 mL deionized water and denoted as solution A. Solution B was prepared by dissolving 3.24 g Na_2_CO_3_ in 50 mL deionized water. Lastly, 6 g NaOH was dissolved in 50 mL of deionized water and denoted as solution C. For the synthesis of NiAl-LDH, solutions A and C were added into solution B in dropwise manner while keeping constant pH of 8 by adjusting the addition rate of solution C. The final addition of solution C was 25 mL, which indicates that 3 g of NaOH was ultimately used, and after co-precipitation, the total NiAl-LDH suspension was 180 mL.

To prepare SiO_2_-NiAl-LDH, one third of the resulting aqueous suspension i.e., 60 mL was withdrawn and added with 300 uL of SiCl_4_ (98%), after which the pH of the solution dropped to ca. 6.5. Thereafter, both suspensions were then stirred at 400 rpm for 6 h at room temperature and subsequently placed in an oven at 80 °C and aged for 12 h. After aging, both suspensions were filtered with anhydrous ethanol and deionized water until the pH of the supernatant was neutral. After drying at 80 °C, the samples were ground to powder. 

The dried powder sample without prior addition of SiCl_4_ (pure NiAl-LDH) was then equally divided into two parts, one of which was added with 25.6 mL of cyclohexane and 300 uL SiCl_4_ and stirred on a magnetic stirrer until the solvent evaporated, and the resulting powder was denoted as SiCl_4_-NiAl -LDH.

All three samples were then calcined in a muffle furnace with a heating rate of 2 °C/min up to 500 °C for 2 h, and then reduced in H_2_ atmosphere (99.999%) at 500 °C for 2 h to obtain oxide catalysts, namely H-NiAl-LDO, H-SiO_2_-NiAl-LDO and H-SiCl_4_-NiAl-LDO.

### 2.2. Catalytic Testing

A dry stainless-steel reaction tube was initially filled with quartz wool and sand followed with 0.075 g of catalyst powder. A thermocouple probe was inserted into the reaction tube and its tip was brought into contact with the catalyst bed. Upon loading the reaction tube into the reaction furnace, nitrogen gas was introduced to set the tube pressure to 0.1 MPa and it was heated to 500 °C at 5 °C/min. Upon reaching the desired temperature, hydrogen gas was then introduced to reduce the catalyst at 500 °C for 2 h at 0.1 MPa. Afterwards, hydrogen gas flow was stopped, and nitrogen was re-introduced into the reaction tube while decreasing the temperature to 150 °C. Subsequently, the gas was switched to CO syngas (H_2_/CO = 3:1) or CO_2_ syngas (H_2_/CO = 4/1), both with a flow rate of 65 mL/min and a space speed of 52,000 (mL g^−1^ h^−1^) before the reaction began at 150 °C. The gas analysis was performed with gas chromatograph GC9790PLUS at 50 °C intervals. Two samples were taken at each temperature point. 

In this experiment, the CO, CO_2_ conversion and CH_4_ selectivity were calculated by the following formulas:

(1) $$\mathrm{CO}\;\mathrm{Conversion}\;(\%)=\frac{n_{\mathrm{CO},in}-n_{\mathrm{CO},out}}{n_{\mathrm{CO},in}}\times 100\%,$$

(2) $${\mathrm{CO}}_{2}\;\mathrm{conversion}\;(\%)=\frac{n_{{\mathrm{CO}}_{2},in}-n_{{\mathrm{CO}}_{2},out}}{n_{{\mathrm{CO}}_{2},in}}\times 100\%,$$

(3) $${\mathrm{CH}}_{4}\;\mathrm{Selectivity}\;(\%)=\frac{n_{{\mathrm{CH}}_{4},out}}{n_{\mathrm{CO},in}-n_{CO,out}}\times 100\%,$$

### 2.3. Catalyst Characterization

X-ray diffraction (XRD) is mainly used to determine the crystal phase and structure, which was performed on D8 advance from Bruker, Ettingen, Germany, using Cu Kα as the radiation source, voltage 45 KV, and current 40 mA.

Scanning electron microscope (SEM) SU8010 (Hitachi, Tokyo, Japan) was used to analyze the morphology of the catalysts.

Transmission electron microscope (TEM) FEI Tecnai G2 F30 (FEI, Hillsboro, OR, USA) was also used for morphology as well as structure and composition analysis.

Temperature-programmed reduction (H_2_-TPR) is to analyze the reduction profile of the active species in the catalyst by H_2_, from which the interactions between the active metal and the support can be determined. The instrument model was Autochem II2920 (Micromeritics, Norcross, GA, USA).

XPS (Thermo ESCALAB 250XI, Waltham, MA, USA) was used to analyze the valence state and binding energy of elements in catalyst samples.

BET analyzer (ASAP 2460), was used to determine the specific surface area, pore volume, pore size and pore distribution of the sample.

The elemental analysis of the sample was determined by ICP (Agilent Co., Ltd., Santa Clara, CA, USA, ICPOES730).

## 3. Results and Discussion

**Figure 1:**
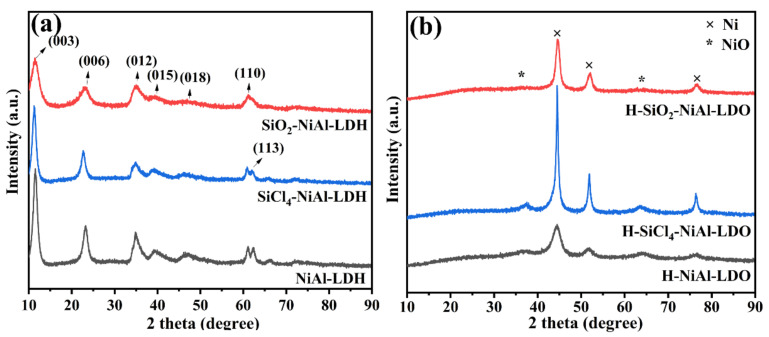
X-ray diffraction patterns of (**a**) NiAl-LDH, SiO_2_-NiAl-LDH, and SiCl_4_-NiAl-LDH and (**b**) H-NiAl-LDO, H-SiO_2_-NiAl-LDO, and H-SiCl_4_-NiAl-LDO.

Figure 1a shows the XRD (X-ray diffraction) patterns of NiAl-LDH and the two catalyst precursors SiO_2_-NiAl-LDH and SiCl_4_-NiAl-LDH, where SiCl_4_ was added to NiAl-LDH in water and cyclohexane as solvent, respectively. The NiAl-LDH sample was found to exhibit good hydrotalcite structure with distinct (003), (006), (009), (015), (110) and (113) crystal planes, typical characteristic peaks of hydrotalcite, indicating that the NiAl-LDH catalyst precursor was successfully prepared. SiCl_4_-NiAl-LDH displays all of the characteristic peaks of hydrotalcite albeit having slightly lower intensity, indicating that the deposition of SiCl_4_ on the NiAl-LDH powder does not significantly affect the crystallinity of the sample. In addition, the characteristic peaks of SiO_2_ were absent, indicating highly dispersed SiO_2_ particles [33]. On the contrary, SiO_2_-NiAl-LDH exhibits lower crystallinity as shown by the broadening of the characteristic peaks.

Figure 1b shows the XRD spectra of the oxide catalysts after the H_2_-programmed reduction treatment (TPR). The reduced catalysts exhibited characteristic peaks at 2θ of 44.51, 51.84, and 76.37°, corresponding to the (111), (200), and (220) crystallographic planes of Ni (JADPS #04-0850), respectively, and at 37.25 and 62.88°, corresponding to the (111) and (200) crystallographic planes of NiO (JADPS #47-1049). Weak diffraction peaks for NiO were present in all of the catalysts, suggesting that a small proportion of NiO was not completely reduced, possibly due to exposure to air when the sample was extracted after reduction. No diffraction peaks of Al_2_O_3_ or other AlO_x_ species were observed in all of the catalysts, which may have been due to the amorphous phase of Al_2_O_3_.

**Figure 2:**
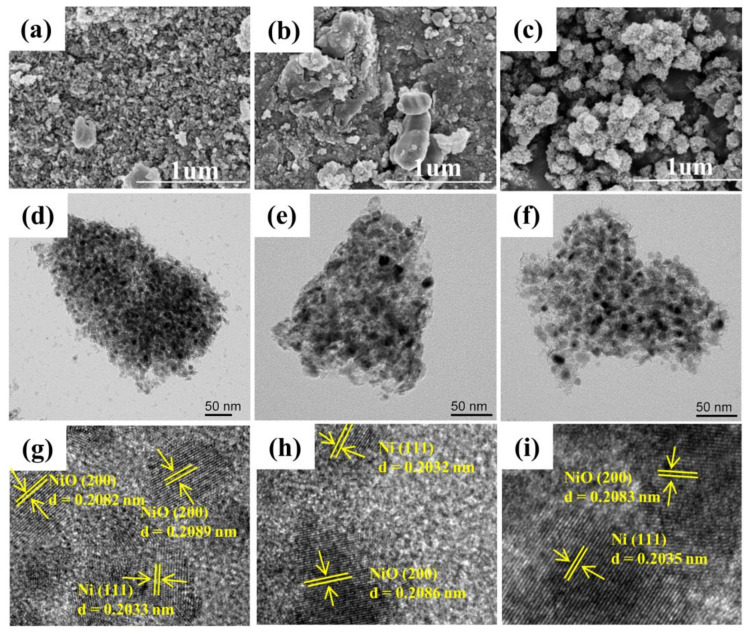
Scanning electron microscopy images of (**a**) H-NiAl-LDO, (**b**) H-SiCl_4_-NiAl-LDO, and (**c**) H-SiO_2_-NiAl-LDO; Transmission electron microscopy (TEM) images of (**d**) H-NiAl-LDO, (**e**) H-SiCl_4_-NiAl-LDO and (**f**) H-SiO_2_-NiAl-LDO and High-resolution TEM images of (**g**) H-NiAl-LDO, (**h**) H-SiCl_4_-NiAl-LDO, and (**i**) H-SiO_2_-NiAl-LDO.

From the SEM (Scanning electron microscopy) images in Figure 2, H-NiAl-LDH catalyst exhibited irregular nanoparticles with considerable aggregation, which may greatly reduce the utilization of the catalyst as only a fraction of the active metals on the catalyst surface can be in contact with the reaction gas. Similarly, extensive agglomeration was also observed for H-SiCl_4_-NiAl-LDO sample, which hardly shows the typical plate-like hydrotalcite morphology. In contrast, H-SiO_2_-NiAl-LDO sample demonstrated well dispersed nanospheres which constituted assemblies of small nanosized particles. This morphology would promote better exposure of the active sites as it may increase the probability of reduction of NiO to the active metal Ni.

Figure 2d–f shows the transmission electron microscopy (TEM) images of the reduced catalysts. Similar to the SEM images, particles agglomeration was observed for H-NiAl-LDO and H-SiCl_4_-NiAl-LDO catalysts, whereas H-SiO_2_-NiAl-LDO showed better dispersion with only a few small agglomerates. The low degree of agglomeration in the latter catalyst suggested that the addition of SiCl_4_ to the aqueous solution during the material’s synthesis was effective in inhibiting the particles aggregation, which led to a better dispersion of the catalyst and impeded the deactivation of the catalyst at high temperatures, thereby improving the catalytic activity.

**Figure 3:**
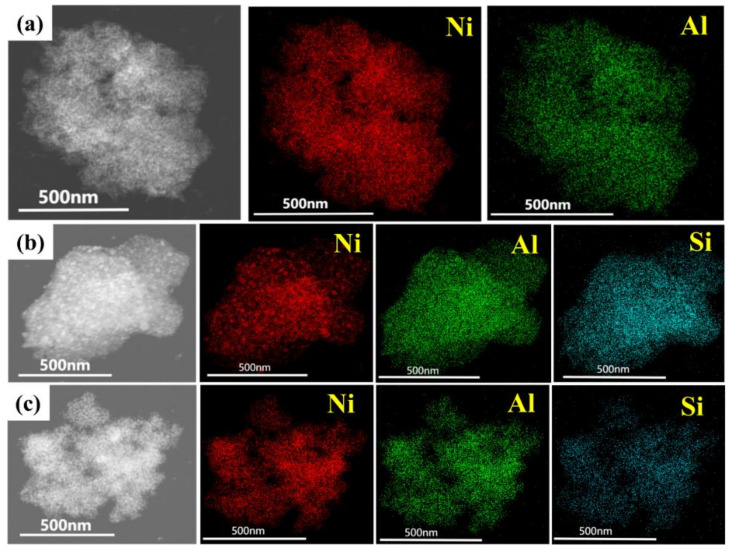
TEM-energy dispersive spectroscopy (TEM-EDS) images of (**a**) H-NiAl-LDO (Reprinted with permission from [34], Copyright 2022 Elsevier), (**b**) H-SiCl_4_-NiAl-LDO, (**c**) H-SiO_2_-NiAl-LDO.

The high-resolution (HR) TEM images of the reduced catalysts (Figure 2g–i) show the lattice arrangement of the (200) crystal plane of NiO and (111) crystal plane of Ni, with lattice spacings of approximately 0.2090 and 0.2035 nm, respectively, which correlate well with the XRD patterns. The TEM energy dispersive spectroscopy (EDS) results in Figure 3 showed uniform dispersion of Ni, Al and Si elements in all of the reduced catalysts [34].

**Figure 4:**
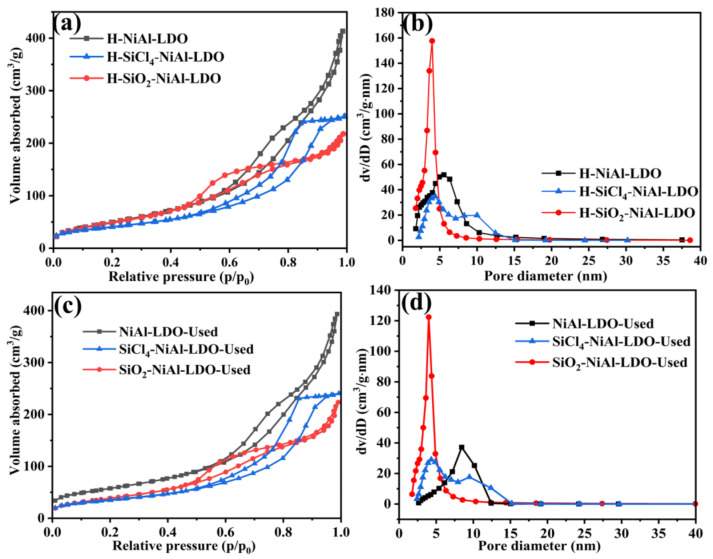
Nitrogen adsorption-desorption isotherms of (**a**) reduced samples and (**c**) used samples; Pore size distribution of (**b**) reduced samples and (**d**) used samples.

To determine the structural properties of the catalysts, we performed N_2_ adsorption–desorption and pore size distribution tests on the reduced catalysts. As seen in Figure 4a, all of the catalysts exhibited type-IV isotherms, which indicated the existence of uniform cylindrical pores. In particular, H-NiAl-LDO and H-SiO_2_-NiAl-LDO catalysts exhibited H3-type hysteresis loops, which were generally associated to the layered structural aggregates and mesoporous or macroporous materials. On the other hand, H-SiCl_4_-NiAl-LDO catalyst exhibited H2-type hysteresis loops, indicating the presence of mesoporous structure. As the catalytic reaction occurred on the catalyst surface, a higher specific surface area is generally more favorable as it renders more active sites. Neither the isotherm nor the hysteresis loop type of the used catalyst changed. However, after high temperature reaction, the catalyst skeleton collapsed and the smaller pores disappeared to form larger pores, so the used catalysts had larger pore sizes than fresh catalysts.

According to Table 1, the specific surface area of sample H-NiAl-LDO, H-SiO_2_-NiAl-LDO and H-SiCl_4_-NiAl-LDO are157, 210, and 147 m^2^/g, respectively. The results show that the addition of SiCl_4_ into the aqueous suspension during the synthesis of NiAl-LDH greatly increases the specific surface area of the catalyst, owing to less particle aggregation as shown by SEM and TEM images. By observing the specific surface area, pore diameter, and pore volume of the catalysts prior to and after the catalytic test, it was observed that the specific surface area of the spent catalysts decreased, which may have been due to the sintering of the catalysts [35].

As shown in Figure 4b, the pore sizes of the reduced catalysts are in the range of 2–15 nm, among which H-NiAl-LDO and H-SiCl_4_-NiAl-LDO have larger pore sizes, 6.72 nm and 7.21 nm, respectively, while that of H-SiO_2_-NiAl-LDO catalyst is 4.98 nm, suggesting the effect of the addition of SiCl_4_ on the pore size of the catalyst. In particular, it can be observed that there are two maxima in the pore size distribution of the H-SiCl_4_-NiAl-LDO catalyst (Figure 4b). The SEM image of this sample (Figure 2b) indicates that the particles in general exhibit two morphologies: small granular material at the bottom of the picture and a large oval-shaped spheres, which may have different pore sizes, resulting in two maxima in the pore size distribution. This could also imply that the addition of SiCl_4_ using cyclohexane as a solvent does not yield a homogeneous mixture, as can be seen in the SEM image where there are larger particles in some areas of the catalyst, which could be due to the smaller interaction between the hydrotalcite and SiCl_4_.

**Figure 5:**
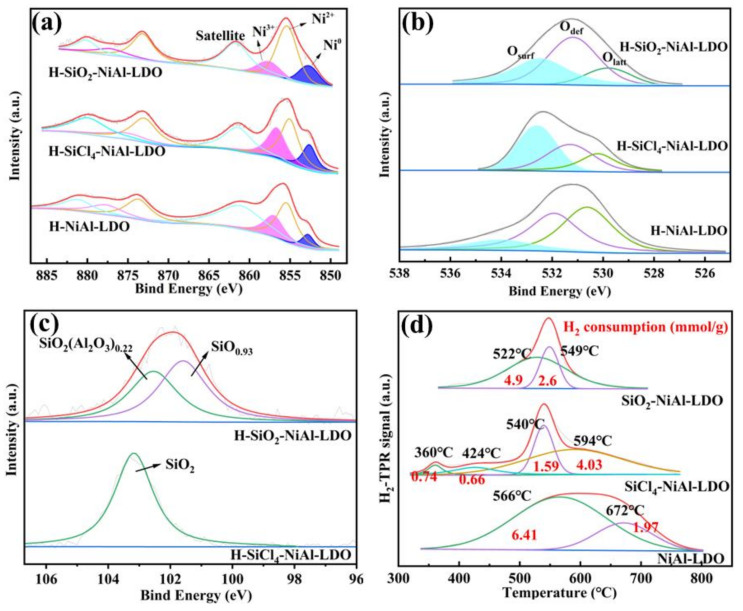
(**a**) Ni 2p XPS spectra, (**b**) O 1s XPS spectra, (**c**) Si 2p XPS spectra and (**d**) H_2_-TPR profiles of H-NiAl-LDO, H-SiCl_4_-NiAl-LDO and H-SiO_2_-NiAl-LDO catalysts.

To explore the surface composition and chemical valence state of each element in the catalyst and the effect of SiCl_4_ on the oxygen vacancies, we conducted X-ray photoelectron spectroscopy (XPS). As shown in Figure 5a, the XPS spectrum of Ni was divided into two spin orbitals, 2p_1/2_ and 2p_3/2_. The center position of the peak at 852.8 eV corresponded to Ni^0^, which is the active metal Ni monomer after H_2_ reduction, the amount of which has an important influence on the catalytic activity. The center position at 855.4 and 873.7 eV corresponded to Ni^2+^, which may be due to the fact that the H_2_ reduction at 500 °C may not be sufficient to oxidize the difficult-to-reduce Ni-Al oxides to Ni^0^ and that it is difficult to avoid some Ni being oxidized during the non-in situ XPS characterization [36]. The center position at 857 and 877.7 eV corresponded to Ni^3+^, possibly originating from Ni_2_O_3_ [37]. A satellite peak with relatively higher binding energy can also be observed next to Ni^3+^ [38]. The binding energy of Ni 2p may change due to the different interaction forces of the components between the different catalysts. The proportion of Ni^0^ and oxygen species for different catalysts are summarized in Table 2. It can be seen that H-SiO_2_-NiAl-LDO catalyst had the highest proportion of Ni^0^, which indicate facile reduction of the NiO species under H_2_ atmosphere to produce active metal for the methanation reaction. In contrast, pure H-NiAl-LDH is relatively stable, where Ni interacts more strongly with the support, thus rendering reduction difficult [39]. In summary, the Ni 2p orbital spectra of the reduced catalysts showed that the binding energy between Ni and the support was decreased by the addition of SiCl_4_ into the aqueous synthesis suspension to produce SiO_2_, thereby allowing NiO species to be reduced at a lower reduction temperature [40], as demonstrated in the subsequent H_2_-TPR characterization. 

Figure 5b compares the XPS spectra of O1s on the surface of the reduced catalysts. The main peak can be divided into three subsidiary peaks, where the peaks near 530.1, 531.2, and 532.6 eV corresponded to the lattice oxygen (O_latt_), defect oxygen (O_def_), and surface adsorption oxygen (O_surf_), respectively. It is apparent from the graphs that the peaks for both H-SiO_2_-NiAl-LDO and H-SiCl_4_-NiAl-LDO catalysts are clearly shifted towards the ground binding energy, indicating a weakening of the interaction forces between their active centers and the carriers, allowing NiO to be reduced to Ni^0^ at lower temperatures. As summarized in Table 2, H-SiO_2_-NiAl-LDO catalyst had the highest ratio of surface adsorbed oxygen at 37% and the lattice oxygen ratio of 15%. The latter was attributed to a metal–oxygen bond with a relatively stable structure. The former was due to the presence of water on the catalyst surface [41], which was more active than the latter, thereby facilitating the reduction process and further influencing the generation of active metals [42], which eventually promotes higher reactivity. This also suggested that the H-SiO_2_-NiAl-LDO catalyst had a greater variety of surface hydroxyl groups, which facilitated better adsorption of CO gas during catalysis and the facile formation of intermediate bicarbonate salts and formate substances during the reaction [41]. On the other hand, the lattice oxygen ratio of H-SiCl_4_-NiAl-LDO catalyst was found to be significantly higher, therefore, making it harder to be reduced.

By analyzing the characteristic peaks of Si in the XPS spectra of the two samples, we found that Si was still present as SiO_2_ in the H-SiCl_4_-NiAl-LDO Catalyst with SiCl_4_ added with cyclohexane as the solvent. This suggests that the catalyst may contain Si–O–Al bonds, which may account for its improved performance [43]. While Si spectra was divided into two peaks in the H-SiO_2_-NiAl-LDO Catalyst with SiCl_4_ added with water as the solvent, corresponding to two compounds, SiO_2_(Al_2_O_3_)_0.22_ and SiO_0.92_, as shown in Figure 5c [44,45]. This suggests that the catalyst may contain Si-O-Al bonds, which may account for its improved performance.

We employed H_2_-TPR characterization to assess the reduction properties of the catalyst and the stability between the active component and support. According to the reduction temperature of each catalyst, it was inferred that the addition of SiCl_4_ in different solvents affected the interaction between Ni particles and the support. From Figure 5d, the reduction peak can generally be divided into three stages. The temperature below 450 °C is α-NiO, which belongs to the free NiO on the catalyst surface. The presence of this peak indicates weak interaction between the active metal and the support. The reduction temperature between 450 and 700 °C (middle-temperature region) can be divided into β1-NiO (450–550 °C), which belongs to the Ni-rich phase that is easily reduced, and β2-NiO (550–700 °C), which belongs to the Al-rich phase that was difficult to be reduced [46].

Figure 5d shows that the hydrogen consumption peaks of the NiAl-LDO catalyst were mainly located in the β2-NiO region (566 °C and 672 °C), thus can be hardly reduced. Both reduction peak temperatures are attributed to the Al-rich orthoclinic region, and as NiO is better reduced than Al_2_O_3_, the material reduced by the higher temperature reduction peak contains more Al. It can be clearly seen that in both reduction peaks, the substance containing more Ni consumes more H_2_, which means that the NiAl-LDO catalyst also contains more of N-rich orthoclinic. The SiCl_4_-NiAl-LDO catalyst exhibited a small reduction peak at 360 °C and 424 °C, which can be ascribed to free NiO particles located on the oxide surface [47]. Higher reduction temperature was observed at 540 °C and 594 °C, indicating the presence of both β1-NiO and β2-NiO. On the other hand, the reduction temperature peaks of the SiO_2_-NiAl-LDO catalyst were located at 522 °C and 549 °C, with higher H_2_ consumption at the former temperature, indicating that most of the NiO in the SiO_2_-NiAl-LDO catalyst was highly reducible to Ni^0^ as they belonged to the β1-NiO region with more Ni-rich phase. Compared with the NiAl-LDO catalyst, the reduction temperature of the SiO_2_-NiAl-LDO catalysts was significantly lower. The H_2_ was consumed at temperatures below 550 °C, indicating that the catalyst was easily reduced, whereby more metallic Ni was generated, which plays an important role as an active component for the methanation reaction [48,49].

Table 3 presents the quantitative analysis of the catalysts as given by inductively coupled plasma (ICP), showing the mass percentage of each element. Using the same amount of SiCl_4_, it was found that more Si was attached to the H-SiCl_4_-NiAl-LDO catalyst, this may be due to the addition of deionized water to the H-SiO_2_-NiAl-LDO catalyst during stirring and ageing, resulting in the loss of some of the SiCl_4_. However, the active metal content is lower than that of H-SiO_2_-NiAl-LDO, indicating that adding SiCl_4_ to water can retain more active components than cyclohexane. By comparing the Ni contents of the H-NiAl-LDO and H-SiO_2_-NiAl-LDO catalysts, it was observed that the H-SiO_2_-NiAl-LDO catalysts contained less active Ni metal but achieved better performance, which may have been because the H-SiO_2_-NiAl-LDO catalyst had a relatively high proportion of Ni^0^. The proportion of active metals in the catalytic process was also relatively high; therefore, a better catalytic effect can be obtained with a relatively small content of Ni.

**Figure 6:**
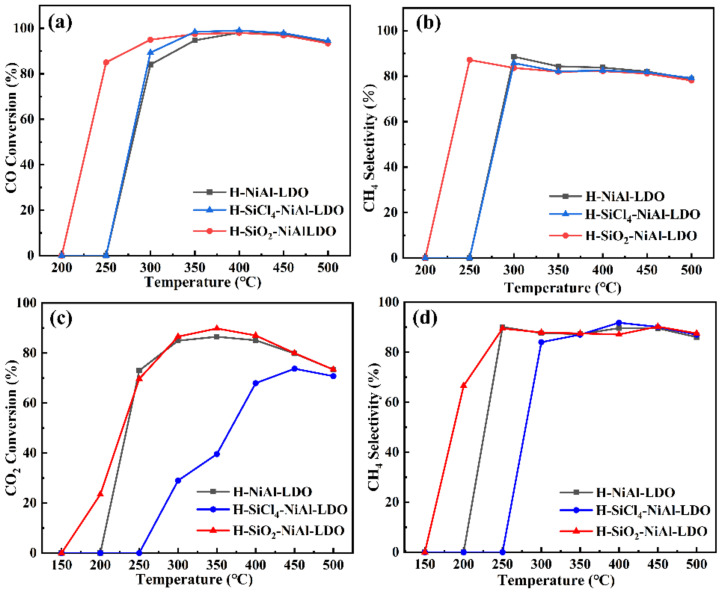
Catalytic performances of catalysts: (**a**) CO conversion, (**b**) CH_4_ selectivity (CO); (**c**) CO_2_ conversion, (**d**) CH_4_ selectivity (CO_2_).

Finally, we tested the catalytic performances of the reduced catalysts for the methanation of CO/CO_2_ and observed that the addition of SiCl_4_ in different solvents had different effects on catalyst performance. As shown in Figure 6, in general, the conversion of each catalyst increased with an increase in temperature. The optimum temperature was found to be around 300 to 400 °C, beyond which (c.a. 450 °C), the conversion of the catalyst decreased, which could be due to sintering of the catalysts at higher temperature. With the space velocity of 52,000 mL·g^−1^·h^−1^, the CO conversion of the H-SiO_2_-NiAl-LDO catalyst reached 85% at 250 °C and 95% at 300 °C. As a comparison, the activity of the H-NiAl-LDO and H-SiCl_4_-NiAl-LDO catalysts only began at 300 °C, with lower CO conversion of 84 and 89%, respectively. For CO_2_ methanation, H-SiO_2_-NiAl-LDO catalyst showed activity at lower temperature of 200 °C, with CO_2_ conversion of 23%. The maximum conversion of 90% was achieved at 350 °C. As a comparison, H-NiAl-LDO and H-SiCl_4_-NiAl-LDO catalysts only started to react after 250 °C. While H-NiAl-LDO could achieve similar CO_2_ conversion of 85% at 350 °C, H-SiCl_4_-NiAl-LDO exhibited much lower conversion of 40% at the same temperature. 

**Figure 7:**
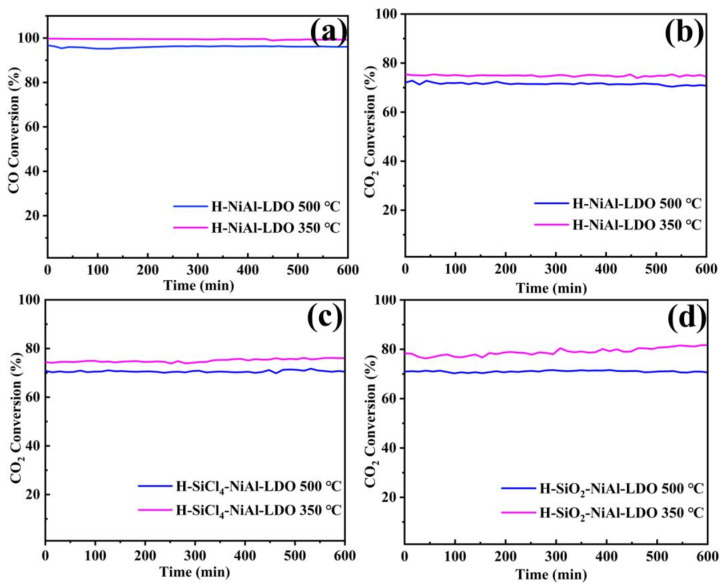
(**a**–**d**) The 10 h CO/CO_2_ methanation performance of each catalyst.

The conversion of each catalyst increases with temperature, and when the temperature reaches a certain point, performance decreases significantly. There are two possible reasons for this phenomenon, which could be the effect of the time of the catalyst reaction on the catalyst performance, or it could be the collapse of the catalyst pores due to high temperatures, resulting in carbon build-up and sintering. In order to provide evidence for this phenomenon, we found that various Ni-based catalysts for CO and CO_2_ methanation have been shown in previous studies to suffer from reduced performance at high temperatures [50,51,52,53]. We then tested the stability of the three catalysts in this study at 350 °C and 500 °C for 10 h (Figure 7) to demonstrate that the performance degradation after 450 °C was due to high temperature catalyst sintering rather than the timing of the catalyst reaction. For CO_2_ methanation reaction we tested all three catalysts and for CO we chose a typical H-NiAl-LDO catalyst. It was observed that there was no decrease in stability for 10 h at the reaction temperature used for either catalyst, and the hydrotalcite catalysts generally had excellent stability, while the conversion at 500 °C was lower than at 350 °C. Therefore, the drop in performance after 450 °C is not related to the stability of the catalysts.

Overall, the results suggest there is different interactions between SiO_2_ and the NiAl-LDH support, depending on the preparation method, whereby the introduction of SiCl_4_ in the aqueous synthesis suspension significantly improved the low-temperature activity of the catalyst for CO and CO_2_ methanation by minimizing the particles agglomeration, increasing specific surface area, tuning the reducibility of the NiO species, and increasing the surface adsorption oxygen. Lastly, Table 4 shows the comparison of the catalytic activity of the prepared catalyst (H-SiO_2_-NiAl-LDO) with those of reported Ni-based catalysts. Given that higher space velocity and lower reaction temperature were used in this study, it can be concluded that the addition of SiCl_4_ into NiAl-LDH to form SiO_2_ has exhibited good low-temperature catalytic performance.

## 4. Conclusions

The effect of treating nickel-aluminum hydrotalcite-derived oxides with SiCl_4_ in water and cyclohexane on the catalyst formation was studied and the effect of SiCl_4_ addition was explored for the first time in the application of Ni-based hydrotalcite for methanation reaction. The performance rankings of the prepared catalysts in this study are: H-SiO_2_-NiAl-LDO > H-NiAl-LDO > H-SiCl_4_-NiAl-LDO, whereby H-SiO_2_-NiAl-LDO demonstrates greater performance in low-temperature methanation reaction. This could be attributed to several factors, such as better particle dispersion and low agglomeration, which leads to larger specific surface area. In addition, high reducibility of the NiO species also increased the catalytic activity as shown by lower reduction temperature under H_2_ environment, suggesting more metallic Ni was generated as the active species. In summary, the addition of SiCl_4_ to form SiO_2_ has a promoting effect on the low temperature performance of CO/CO_2_ methanation. This method of introducing SiCl_4_ into water to form SiO_2_ to modify NiAl-LDO to improve CO/CO_2_ methanation performance provides a strategy for the modification of other metal catalysts. In addition, further fine tuning, such as varying the amount of SiCl_4_ as well as adjusting the Ni/Al molar ratio may further change the particles size, surface area, and aggregation state, which will eventually affect the catalytic activity of the catalysts.

## Figures and Tables

**Table 1:** Textural properties of the prepared catalysts.

Samples	S_BET_ (m^2^·g^−1^)	D_BJH_ (nm)	Vp (cm^3^·g^−1^)
NiAl-LDO	157	6.72	0.38
SiCl_4_-NiAl-LDO	147	7.21	0.38
SiO_2_-NiAl-LDO	210	4.98	0.34
NiAl-LDO-Used	114	8.37	0.35
SiCl_4_-NiAl-LDO-Used	132	7.65	0.37
SiO_2_-NiAl-LDO-Used	163	6.30	0.34

**Table 2:** Quantification of the metallic Ni and oxygen species of the prepared catalysts.

Catalysts	Ni^0^/(Ni^0^ + Ni^2+^ + Ni^3+^)	O_surf_/(O_surf_ + O_def_ + O_latt_)	O_latt_/(O_surf_ + O_def_ + O_latt_)
H-NiAl-LDO	0.13	0.12	0.49
H-SiCl_4_-NiAl-LDO	0.19	0.22	0.44
H-SiO_2_-NiAl-LDO	0.21	0.37	0.15

**Table 3:** Elemental analysis of the prepared catalysts.

Samples	H-NiAl-LDO	H-SiCl_4_-NiAl-LDO	H-SiO_2_-NiAl-LDO
Ni (wt%)	60.62	51.33	53.31
Al (wt%)	10.43	5.70	7.29
Si (wt%)	0	4.70	3.02

**Table 4:** Comparison of low temperature catalysts prepared via different methods.

Catalysts	WHSV (mL·g^−1^·h^−1^)	T (°C)	CO_2_ Conversion (%)	Ref
15% Ni/Al_2_O_3_ (Plasma)	8700	250	40	[54]
Ni/SBA-15	15,000	250	82	[55]
Ni-sepiolite	36,000	250	10	[56]
Ni/La_2_O_2_CO_3_	20,000	300	25	[57]
Ni20Fe1.5-LDH	12,000	250	76	[58]
Ni/MgAl-LDH	5017	250	20	[25]
NaY-Ni5-LDHs	30,000	250	13	[59]
H-SiO_2_-NiAl-LDO	52,000	200	23	This study

## Data Availability

The data is available on reasonable request from the corresponding author.
